# Correlation between dental and skeletal maturity in Korean children based on dental maturity percentile: a retrospective study

**DOI:** 10.1186/s12903-024-04015-0

**Published:** 2024-03-22

**Authors:** Seung-Hwan Ong, Hyuntae Kim, Ji-Soo Song, Teo Jeon Shin, Hong-Keun Hyun, Ki-Taeg Jang, Young-Jae Kim

**Affiliations:** Department of Pediatric Dentistry, School of Dentistry, Seoul National University, 101 Daehak-ro, Jongno-gu, Seoul, 03080 Korea

**Keywords:** Dental maturity, Skeletal maturity, Dental maturity percentile, Demirjian method, Cervical vertebral maturation method

## Abstract

**Background:**

The correlation between dental maturity and skeletal maturity has been proposed, but its clinical application remains challenging. Moreover, the varying correlations observed in different studies indicate the necessity for research tailored to specific populations.

**Aim:**

To compare skeletal maturity in Korean children with advanced and delayed dental maturity using dental maturity percentile.

**Design:**

Dental panoramic radiographs and cephalometric radiographs were obtained from 5133 and 395 healthy Korean children aged between 4 and 16 years old. Dental maturity was assessed with Demirjian’s method, while skeletal maturity was assessed with the cervical vertebral maturation method. Standard percentile curves were developed through quantile regression. Advanced (93 boys and 110 girls) and delayed (92 boys and 100 girls) dental maturity groups were defined by the 50th percentile.

**Results:**

The advanced group showed earlier skeletal maturity in multiple cervical stages (CS) in both boys (CS 1, 2, 3, 4, and 6) and girls (CS 1, 3, 4, 5, and 6). Significant differences, as determined by Mann-Whitney U tests, were observed in CS 1 for boys (*p* = 0.004) and in CS 4 for girls (*p* = 0.037). High Spearman correlation coefficients between dental maturity and cervical vertebral maturity exceeded 0.826 (*p* = 0.000) in all groups.

**Conclusion:**

A correlation between dental and skeletal maturity, as well as advanced skeletal maturity in the advanced dental maturity group, was observed. Using percentile curves to determine dental maturity may aid in assessing skeletal maturity, with potential applications in orthodontic diagnosis and treatment planning.

**Supplementary Information:**

The online version contains supplementary material available at 10.1186/s12903-024-04015-0.

## Introduction

Assessment of dental and skeletal maturity is important for establishing an orthodontic treatment plan for a growing child with malocclusion [1, 2]. The timing of orthodontic treatment using functional and orthopedic appliances to promote proper skeletal growth varies depending on the type of malocclusion, and the necessity and timing of tooth extraction should also consider dentofacial growth and development [3, 4]. Therefore, it is essential to evaluate and consider each child’s dentofacial growth and development for optimal orthodontic treatment results. To evaluate patient growth, physiologic indicators, such as sexual, skeletal, and dental maturity are preferred, as chronological age by birth date exhibits wide individual variation and cannot be considered a reliable indicator for maturity [5]. In dentistry, dental and skeletal maturity are widely used indicators for assessing growth [6].

Dental development serves as a valuable indicator of maturation because it is less influenced by environmental factors and exhibits high reliability with a low coefficient of variation [7]. The assessment of dental development based on panoramic radiographs is the most commonly used method in developing dentition, and the method proposed by Demirjian is considered a representative approach [7, 8]. Demirjian’s method classifies seven permanent teeth of the left mandible into eight developmental stages, and the maturity score is determined by summing the scores assigned to each tooth’s development stage [9]. This calculated maturity score can be used to gauge the participant’s progress in dental maturation or be converted into dental age. Additionally, if standard maturation score data within a given population are available, the relative speed of a participant’s dental maturation can be evaluated [10].

Widely used methods for evaluating skeletal maturity include assessing cervical vertebrae using lateral cephalometric radiographs and examining hand-wrist bones from hand-wrist radiographs [5]. The cervical vertebral maturation (CVM) method proposed by Baccetti et al. classifies bone maturation in growing children into six stages based on the maturity of the 2nd, 3rd, and 4th cervical vertebrae, and enables clinicians to identify optimal timing for the treatment of dentofacial orthopedics treatment [3]. Given concerns about radiation exposure from radiographs, the cervical vertebrae method offers the advantage of reducing additional radiation exposure compared to the hand-wrist method. The cervical vertebrae method uses cephalometric radiographs, which are routinely taken for orthodontic diagnosis and treatment planning, and show similar simplicity, objectivity, repeatability and validity results as the hand-wrist method [2, 3].

Several studies have been conducted on the correlation between dental maturity and bone maturity, but a consensus has not been reached. As both dental maturity and bone maturity increase with age, they show a certain degree of correlation, but the degree varies among studies. Morris [1], Chen [2], Jeong [11], and Bittencourt [12] reported a strong correlation between dental maturity and skeletal maturity, Demirjian [13] reported a low correlation, and Uysal [14] and Krailassiri [15] found different correlations for individual teeth. Dental maturity and skeletal maturity are both affected by genetic and ecological factors [16]. Differences in study designs across various races and populations resulted in varying outcomes among studies.

To apply statistical correlation results in clinical practice to predict skeletal maturity from dental maturity, confirming whether a child has advanced or delayed dental maturity is necessary. However, this confirmation is limited without percentile data within the population, and because dental maturity varies depending on race and ethnicity, the dental maturity percentile of each population should be needed [16]. If the correlation between dental and skeletal maturity can be utilized clinically, it would aid in confirming growth and development with low radiation exposure and help planning orthodontic treatment.

Therefore, this study aimed to establish dental maturity percentile curves for Korean children using Demirjian’s method and to compare skeletal maturity between children with advanced and delayed dental maturity.

## Materials and methods

### Sample collection

This study was designed as a retrospective study, and it was conducted according to the Declaration of Helsinki principles. The study was approved by the Institutional Review Board of the Seoul National University Dental Hospital, Seoul, Korea (Ethics Code: ERI23026). Dental panoramic radiographs were collected from 5133 healthy children (2825 boys and 2308 girls) aged between 4 and 16 years who visited the Seoul National University Dental Hospital Department of Pediatric Dentistry between 2020 and 2021. Cephalometric radiographs were collected from 395 children (185 boys and 210 girls) out of the entire sample who had taken both panoramic and cephalometric radiographs on the same day for orthodontic assessment. Radiographic images were obtained using radiographic machines, including the OP-100 (Imaging Instrumentarium, Tuusula, Finland) and RAYSCAN alpha (Ray Co., Ltd., Hwaseong-si, Korea). The age and sex distributions of the participants are presented in Table 1. All the participants involved in this study were Korean.

**Table 1:** Age and sex distribution of the panoramic and cephalometric radiograph samples

Chronologic age	Panoramic		Cephalometric	
	Boys	Girls	Boys	Girls
4.0–4.99	175	144		
5.0–5.99	286	201		1
6.0–6.99	366	272	4	8
7.0–7.99	365	316	30	41
8.0–8.99	343	287	33	36
9.0–9.99	359	293	32	28
10.0–10.99	311	276	31	24
11.0–11.99	217	191	17	27
12.0–12.99	174	133	17	16
13.0–13.99	111	106	13	19
14.0–14.99	74	53	4	4
15.0–15.99	44	36	4	6
Total	2825	2308	185	210

The exclusion criteria were participants with systemic diseases or genetic disorders that would affect skeletal and dental growth; panoramic radiographs of poor quality; history of orthodontic treatment; history of extraction of permanent teeth and congenitally missing teeth on the left side of the mandible; and localized oral pathology, anomalies, or impacted teeth that would affect dental development.

### Assessment of dental and skeletal maturity

The tooth developmental stages of the left seven mandibular teeth (incisors, canines, premolars, and molars, except third molars) in the panoramic radiographs were evaluated using Demirjian’s method (Supplementary Fig. 1) [9]. Tooth development was divided into eight stages, from A “There is no fusion of these calcified points” to H “The apical end of the root canal is completely closed”. Each stage of the seven teeth was converted into self-weighted scores for boys and girls. The sum of the scores represented the participant’s dental maturity, with a completion score of 100.

The skeletal maturation stage was evaluated with cephalometric radiographs using the cervical vertebral maturation (CVM) method by Baccetti et al. (Supplementary Fig. 2) [3]. Six maturational stages, from cervical stage 1 (CS 1) “The lower borders of all three vertebrae are flat” to cervical stage 6 (CS 6) “The concavities at the lower borders of three cervical vertebrae (C 2, C 3, and C 4) still are evident” were assigned with the morphology of the bodies of the second (C 2), third (C 3), and fourth (C 4) cervical vertebrae.

Demirjian’s method and CVM method for the dental and skeletal maturity analysis were evaluated by a skilled pediatric dentist.

### Establishing dental maturity percentile curves and tables

The chronological age was obtained by subtracting the date of birth from the date the radiographic image was taken and then dividing by 365 to convert it into decimal years.

Standard percentile curves for dental maturity were obtained using fifth-degree quantile regression, with age as the independent variable and score as the dependent variable. Percentile curves and tables were calculated for the 5th, 16th, 50th, 84th, and 95th percentiles.

### Data and statistical analysis

Intraobserver reliability was assessed with weighted Cohen’s kappa analysis using MedCalc® Statistical Software (version 20.100; MedCalc Software Ltd, Ostend, Belgium). The developmental stage of each tooth was re-examined using 200 randomly selected panoramic radiographs, and the CVM stage was re-examined using 50 randomly selected cephalometric radiographs at 3-week intervals. The calculated weighted Cohen’s kappa values were 0.93 for Demirjian’s method and 0.94 for the CVM method, indicating ‘almost perfect’ agreement.

Quantile regression analyses were performed to establish percentile curves for dental maturity using R software (version: 4.2.1; R Foundation for Statistical Computing). The age difference in skeletal maturation stages between the advanced and delayed groups was compared using the Mann-Whitney U test, and the correlation between dental maturity score and skeletal maturation stage was assessed using Spearman’s rank correlation analysis. A *p* value of < 0.05 was considered significant. The Mann-Whitney U test and Spearman’s rank correlation analyses were conducted with SPSS Statistics 25 (IBM Corp, Armonk, NY, USA).

## Results

### Dental maturity percentiles of Korean children using quantile regression

Standard percentile curves and tables for dental maturity were created using quintic quantile regression analysis. Quantile regression equations for the standard curve are as follows:

$$\eqalign{{\rm{Boys}}:\,{\rm{Score}}\, = \, & 225.260\, - \,155.275\,{\rm{x}}\,\left( {{\rm{Age}}} \right) \cr & + \,40.028\,{\rm{ x}}\,{\left( {{\rm{Age}}} \right)^2}\, - \,4.322\,{\rm{x}}\,{\left( {{\rm{Age}}} \right)^3} \cr & + \,0.214\,{\rm{x}}\,{\left( {{\rm{Age}}} \right)^4}\, - \,0.004\,{\rm{x}}\,{\left( {{\rm{Age}}} \right)^5} \cr}$$

$$\eqalign{{\rm{Girls}}:\,{\rm{Score}}\, = \, & 163.338\, - \,128.721\,{\rm{x}}\,\left( {{\rm{Age}}} \right) \cr & + \,36.388\,{\rm{x}}\,{\left( {{\rm{Age}}} \right)^2}\, - \,4.131\,{\rm{x}}\,{\left( {{\rm{Age}}} \right)^3} \cr & + \,0.212\,{\rm{x}}\,{\left( {{\rm{Age}}} \right)^4}\, - \,0.004\,{\rm{x}}\,{\left( {{\rm{Age}}} \right)^5} \cr}$$

Age was set as a function of the score, and the coefficient of determination (R²) of the equations was 0.9397 for boys and 0.9407 for girls.

**Fig. 1:**
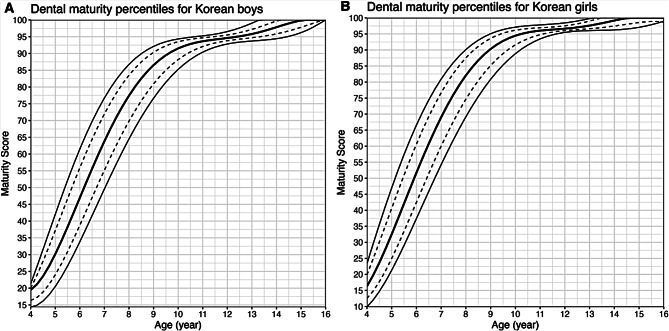
(A) Dental maturity percentiles for Korean boys, (B) Dental maturity percentiles for Korean girls (Bold line: 50th percentile; dotted line: 16th and 84th percentiles; solid line: 5th and 95th percentiles)

The maturity scores and corresponding to the 5th, 16th, 50th, 84th, and 95th percentiles are listed in Supplementary Table 1, and percentile curves are shown in Fig. 1. The age at which the 50th percentile curve reached a maturity score of 100 was 15.25 years for boys and 14.75 years for girls.

### Distribution of participants in the advanced and delayed dental maturity groups

**Fig. 2:**
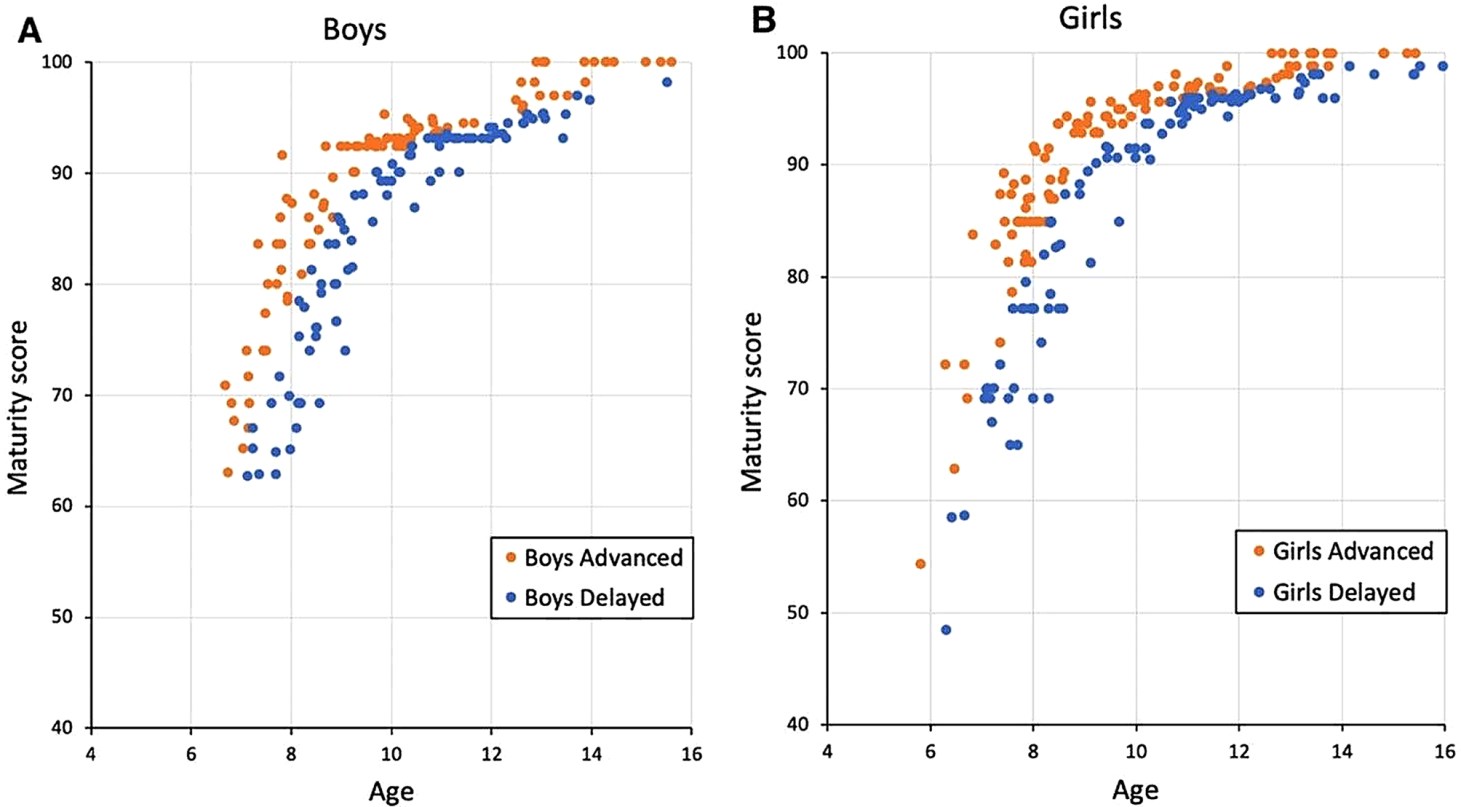
Distribution of the samples in the advanced and delayed dental maturity groups. (A) Boys, (B) Girls

Among 395 children included in the present study, 185 were boys and 210 were girls. Based on the dental maturity percentile curve (Supplementary Table 1 and Fig. 1), 93 boys and 110 girls above the 50th percentile were classified into the ‘A, advanced’ group, and 92 boys and 100 girls below the 50th percentile were classified into the ‘D, delayed’ group (Fig. 2; Table 2). The mean ages at the time of image acquisition were similar in all groups.

**Table 2:** Mean age and dental maturity score of samples in the advanced and delayed groups

	Mean age ± SD	Mean dental maturity score ± SD	n
Boys A	9.96 ± 2.31	89.10 ± 9.28	93
Boys D	10.21 ± 1.87	85.36 ± 10.00	92
Total	10.08 ± 2.10	87.24 ± 9.80	185
Girls A	9.85 ± 2.28	91.57 ± 8.19	110
Girls D	10.26 ± 87.27	87.27 ± 11.50	100
Total	10.04 ± 2.31	89.52 ± 10.11	210

A, advanced; D, delayed; SD, standard deviation

### Skeletal maturity difference between the advanced and delayed groups

The mean ages corresponding to each skeletal maturation stage for each group are presented in Table 3. The advanced group exhibited advanced skeletal maturity in most of the cervical stages compared to the delayed group in both boys and girls. In boys, the advanced group showed younger age at the CS 1, 2, 3, 4, and 6 stages, and in girls, the mean age of the CS 1, 3, 4, 5, and 6 stages was earlier in the advanced group. However, significant differences were observed only in CS 1 for boys (*p* = 0.004) and in CS 4 for girls (*p* = 0.037).

**Table 3:** Mean age and differences between advanced and delayed dental maturity groups according to cervical vertebral maturation stages

Parameter	CS 1		CS 2		CS 3		CS 4		CS 5		CS 6	
	n	Mean age ± SD	n	Mean age ± SD	n	Mean age ± SD	n	Mean age ± SD	n	Mean age ± SD	n	Mean age ± SD
Boys A	16	7.38 ± 0.69	15	8.41 ± 0.85	26	9.43 ± 1.15	22	11.02 ± 1.42	8	13.36 ± 0.62	6	14.56 ± 0.99
Boys D	12	8.11 ± 0.69	16	8.68 ± 0.93	30	9.81 ± 1.15	27	11.63 ± 1.04	6	13.26 ± 0.54	1	15.52
Difference (*p* value)	0.004		NS		NS		NS		NS		NS	
Girls A	12	7.40 ± 0.83	29	8.09 ± 0.85	15	8.69 ± 0.84	33	10.72 ± 1.50	14	12.86 ± 0.99	7	13.73 ± 0.94
Girls D	13	7.59 ± 0.62	14	7.84 ± 0.57	20	9.06 ± 1.14	33	11.13 ± 1.03	14	13.01 ± 1.33	6	14.48 ± 1.07
Difference (*p* value)	NS		NS		NS		0.037		NS		NS	

CS, cervical stage; NS, no statistically significant differences; SD, standard deviation; A, advanced; D, delayed. *p* values from Mann-Whitney U test

### Correlation of dental maturity with skeletal maturity cervical vertebral maturation (CVM) stages

The correlation between dental maturity scores and cervical vertebral maturation stages was assessed in each group, and all groups showed a strong correlation (Table 4). The Girls D group (0.883, *p* < 0.001) showed the highest correlation, while the Boys D group (0.0826, *p* < 0.001) exhibited the lowest correlation.

**Table 4:** Correlations of dental maturity score and cervical vertebral maturation (CVM) stages

	Boys A		Boys D		Girls A		Girls D	
	Correlation	*p* value	Correlation	*p* value	Correlation	*p* value	Correlation	*p* value
CVM	0.868	0.000	0.826	0.000	0.864	0.000	0.883	0.000

A, advanced; D, delayed. *p* values from Spearman’s rank correlation test

## Discussion

For optimal orthodontic treatment, a comprehensive assessment of a patient’s growth and maturation status is essential [17]. However, frequent dental radiography for maturation assessment should be avoided, as clinicians must adhere to the ALADA (As Low As Diagnostically Achievable) principle, which emphasizes minimal radiation exposure [18]. If panoramic radiographs, routinely taken for assessing dental anomalies and development, can provide insights into bone maturity status, they can significantly assist clinicians in obtaining diagnostic information with minimal radiation exposure. Previous studies primarily determined the relationship between dental and skeletal maturity by identifying the specific tooth and developmental stage exhibiting the highest correlation with skeletal maturation for boys and girls respectively [2, 11, 14, 15]. However, the wide variation of age in the developmental stage of individual teeth presents limitations in predicting a child’s skeletal maturation through this statistical correlation in clinical practice.

The purpose of this study was to investigate clinical differences in the relationship between dental maturity and bone maturity by comparing the ages corresponding to skeletal maturity stages in children with advanced and delayed dental maturity. Research on whether differences in dental maturation occur when skeletal maturity varies has been conducted in studies on children with precocious puberty, which reported early maturation of teeth in the precocious puberty group [19, 20]. However, studies on whether differences in dental maturation are associated with variations in skeletal maturity have been limited. There have been studies confirming advanced dental maturity in cases of systemic diseases such as juvenile rheumatoid arthritis [21], but it is difficult to determine whether dental maturity is accelerated in healthy children. To determine whether an individual’s dental maturation is advanced or delayed for their age, the use of dental maturity percentiles is necessary [22]. Since dental maturity varies by race, ethnicity, and over time, it is important to employ percentiles specific to the population at that given time for accurate classification [8, 10, 16]. Furthermore, differences between dental age and chronological age do not necessarily indicate differences in maturity. The estimated dental age, when converted from dental maturity, tends to over/underestimate chronological age, and its accuracy varies between populations and maturity estimation methods [23, 24]. Therefore, since there was no dental maturity percentile curve available for Korean children, this study developed a dental maturity percentile graph and table for Korean children using Demirjian’s method (Fig. 1 and Supplementary Table 1). The participants were then classified as having advanced or delayed dental maturity based on the 50th percentile (Fig. 2).

Demirjian’s original method, which evaluates seven left mandibular teeth for assessing dental maturity, is widely recognized and accepted in forensic science due to its universally applicable maturity scoring system, simplicity, reliable standardization, and excellent reproducibility [7]. Both the tooth calcification stages described by Demirjian and the cervical vertebral maturation method by Baccetti are easy to measure, with substantial intra- and interexaminer reliability [7, 23, 25]. The study utilized dental panoramic radiographs of children who visited Seoul National University Dental Hospital in Korea. Given the ethnically homogeneous nature of the Korean population, the dental maturity scores calculated from this database are likely to represent those of Korean children in general. Quantile regression was employed to establish the standard percentile curve for dental maturity in Korean children. Scores corresponding to the 5th, 16th, 50th, 84th, and 95th percentiles from ages 4 to 16 years are presented in **Supplementary Table 1**. Scores near the 5th percentile indicate delayed dental maturity, while those near the 95th percentile indicate advanced dental maturation. Completion of dental development (maturity score 100) was faster in girls (14.75 years for girls and 15.25 years for boys), consistent with other studies showing that girls exhibit faster dental development than boys [8, 16].

In the present study, the advanced dental maturity group exhibited earlier skeletal maturation than the delayed group in both boys and girls (Table 3). A significant difference was observed in the CS 1 stage for boys and the CS 4 stage for girls. In the remaining cervical stages, no significant differences were observed. However, except for the CS 5 stage in boys and the CS 2 stage in girls, all the cervical stages showed earlier maturation in the advanced group. The earlier maturation of the CS 3 and CS 4 stages observed in the advanced group in both boys and girls, as well as the significant difference in the CS 4 stage in girls, suggests that the relationship between dental and skeletal maturity may have clinical applicability in detecting a pubertal peak in mandibular growth, which is crucial for orthodontic diagnosis and treatment. The CS 3 and CS 4 stages are known as the circumpubertal stage, in which the peak mandibular growth will occur during the year after the CS 3 stage, and the peak in mandibular growth will occur within 1 or 2 years before the CS 4 stage [3, 25]. The overall correlation of the dental maturity score and cervical vertebral maturation stages is shown in Table 4. In both boys and girls, both the advanced group and the delayed group exhibited a significant correlation between dental maturity and skeletal maturity. If the relationship between dental and skeletal maturity becomes more apparent through further studies, dental maturity could be used to complement chronological age in determining the timing of radiography for skeletal maturity assessment. This approach could enable patients to receive optimal orthodontic diagnosis and treatment at the right time while minimizing radiation exposure. The results of this study also emphasize the importance of establishing percentile curves for specific populations, as they can serve as valuable tools for clinicians to assess an individual’s dental maturity relative to their age group in the population and may also find utility in skeletal maturation assessments.

The limitations of this study include the relatively small number of cephalometric samples compared to the entire panoramic sample, as cephalometric radiographs were only taken for orthodontic analysis, and not routinely. Additionally, our study used Demirjian’s method and the CVM method for the evaluation of dental and skeletal maturity. However, since there are various other evaluation methods available, further research is needed to identify the most representative evaluation method that exhibits the strongest correlation with dental and skeletal maturity. Despite these limitations, the results of the study confirmed that the correlation between dental maturity and skeletal maturity could be observed in clinical practice with the help of dental maturity percentiles, and the differences in dental maturity appeared to provide valuable insights into skeletal maturity. Dental maturity may offer a deeper understanding of an individual’s growth and development, extending beyond the assessment of tooth development stages. Therefore, it is imperative for clinicians and forensic odontologists to pay more attention to dental maturity, and further research on growth and development related to dental maturity, with larger sample sizes, will be necessary.

## Conclusion

The current study showed that children with advanced dental maturity tend to exhibit advanced skeletal maturity in both boys and girls. The correlation between skeletal and dental maturity could be utilized in clinical practice with the help of dental maturity percentile data specific to the population. This finding holds potential applications in orthodontic diagnosis and treatment planning, helping to determine the optimal time for radiography and reduce excessive radiation.

### Electronic supplementary material

Below is the link to the electronic supplementary material.

Supplementary Material 1

Supplementary Material 2

Supplementary Material 3

## Data Availability

The data that support the findings of this study are available from the corresponding author, upon reasonable request.

## Abbreviations

CS: cervical stage
CVM: cervical vertebral maturation
