# Supplementary figures and images for: Correlation between dental and skeletal maturity in Korean children based on dental maturity percentile: a retrospective study
Source: BMC Oral Health. 2024 Mar 22;24:377. doi: 10.1186/s12903-024-04015-0 (PMC10958867; doi:10.1186/s12903-024-04015-0)

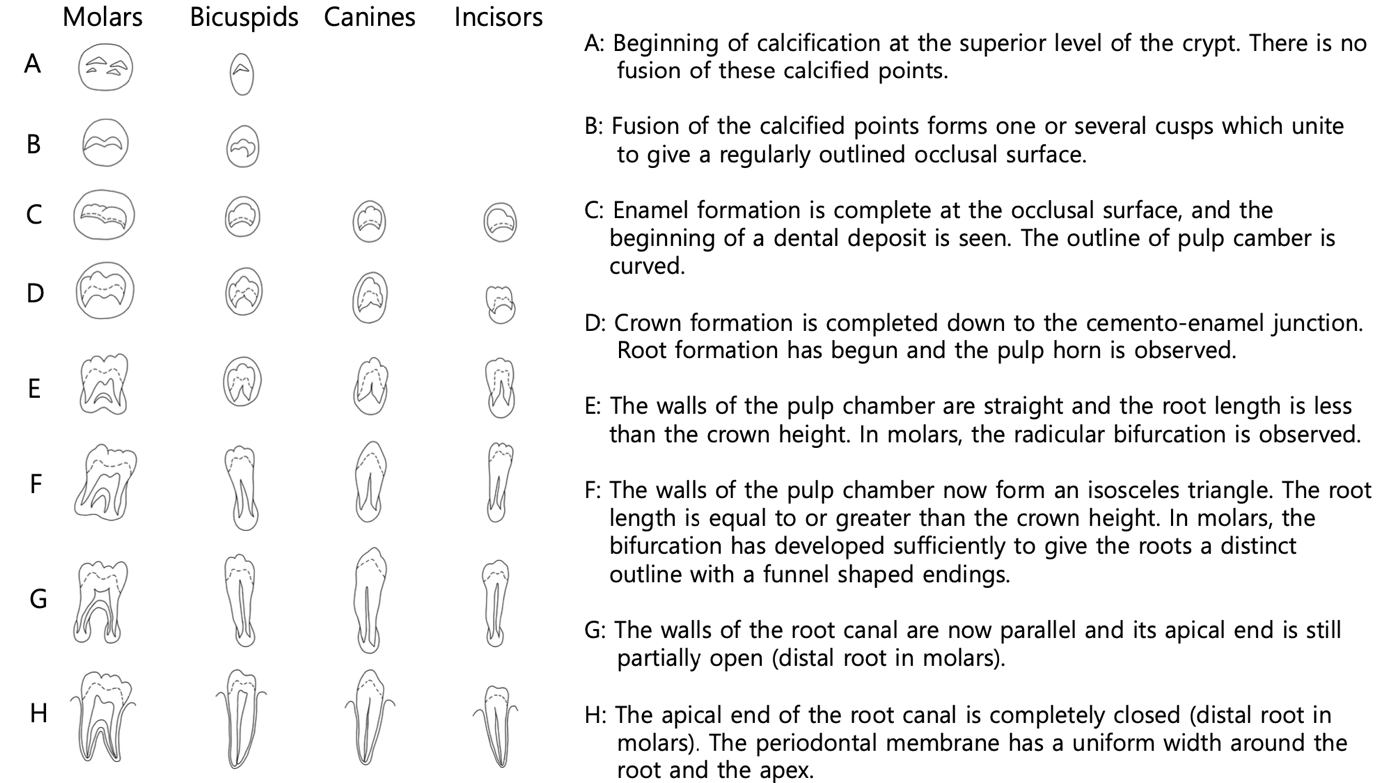


Supplementary Figure 1. The tooth development stages as described by Demirjian et al [9].

Supplement: Supplementary file 1 — Supplementary Material 1 [file 12903_2024_4015_MOESM1_ESM.docx]
