# Supplementary material for: Correlation between dental and skeletal maturity in Korean children based on dental maturity percentile: a retrospective study
Source: BMC Oral Health. 2024 Mar 22;24:377. doi: 10.1186/s12903-024-04015-0 (PMC10958867; doi:10.1186/s12903-024-04015-0)
Supplement: Supplementary file 2 — Supplementary Material 2 [file 12903_2024_4015_MOESM2_ESM.docx]

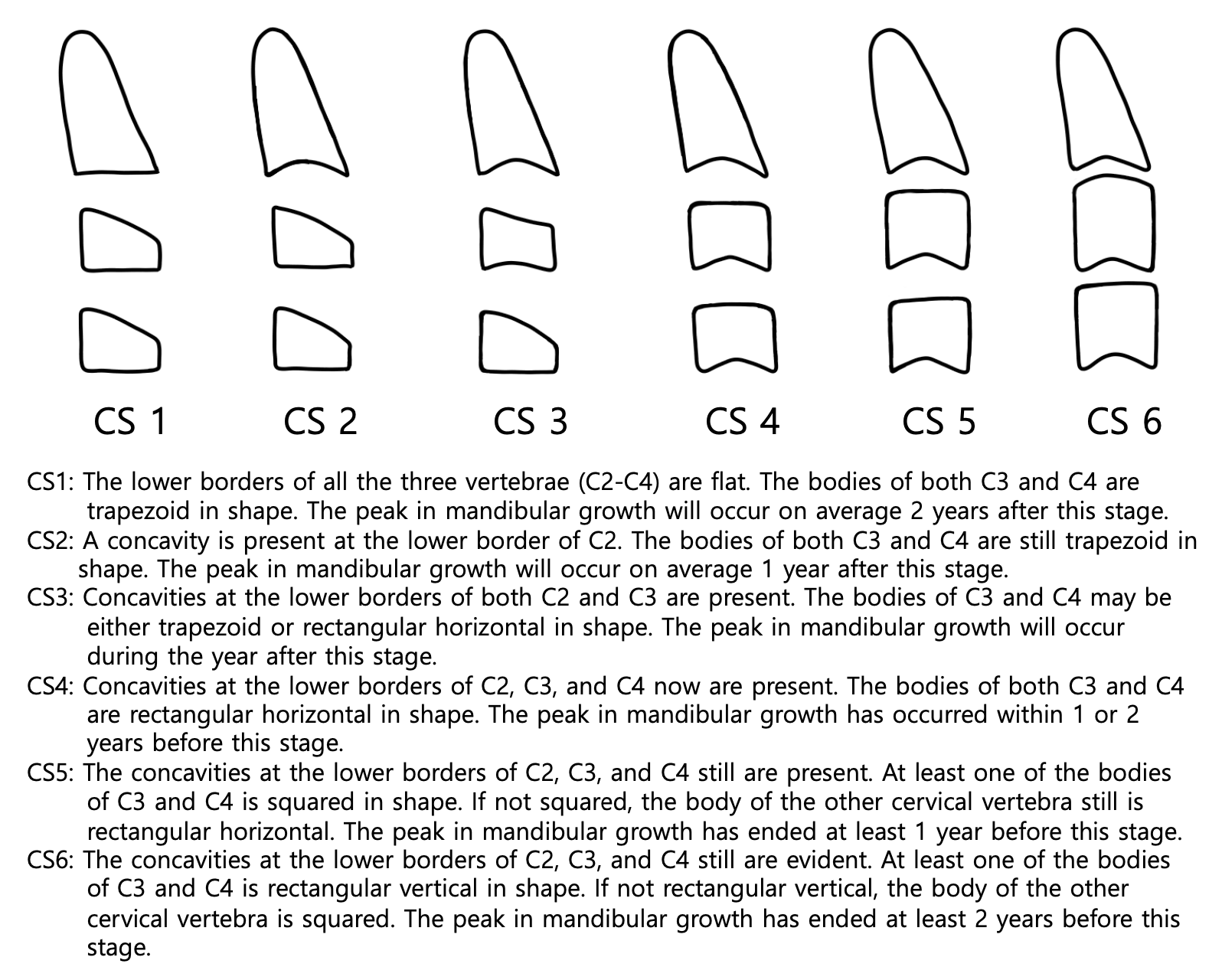


Supplementary Figure 2. The cervical vertebral maturation stages as described by Baccetti et al [3].
