# Supplementary material for: Correlation between dental and skeletal maturity in Korean children based on dental maturity percentile: a retrospective study
Source: BMC Oral Health. 2024 Mar 22;24:377. doi: 10.1186/s12903-024-04015-0 (PMC10958867; doi:10.1186/s12903-024-04015-0)
Supplement: Supplementary file 3 — Supplementary Material 3 [file 12903_2024_4015_MOESM3_ESM.docx]

**Supplementary Table 1** Dental maturity score percentiles A. Boy’s percentile and B. Girl’s percentile

| A. Boy’s Percentile | | | | | | B. Girl’s Percentile | | | | | |
| --- | --- | --- | --- | --- | --- | --- | --- | --- | --- | --- | --- |
| **Age** | **5%** | **16%** | **50%** | **84%** | **95%** | **Age** | **5%** | **16%** | **50%** | **84%** | **95%** |
| **4** | 14.35 | 16.33 | 19.47 | 19.97 | 21.28 | **4** | 9.86 | 12.52 | 16.31 | 19.85 | 23.32 |
| **4.25** | 14.87 | 17.18 | 21.11 | 23.79 | 26.32 | **4.25** | 12 | 14.79 | 19.71 | 24.85 | 29.5 |
| **4.5** | 16.14 | 18.82 | 23.54 | 28 | 31.51 | **4.5** | 14.68 | 17.7 | 23.63 | 30.04 | 35.54 |
| **4.75** | 18.04 | 21.12 | 26.6 | 32.5 | 36.77 | **4.75** | 17.8 | 21.13 | 27.94 | 35.32 | 41.38 |
| **5** | 20.48 | 23.97 | 30.15 | 37.17 | 42.01 | **5** | 21.29 | 24.97 | 32.52 | 40.62 | 46.99 |
| **5.25** | 23.36 | 27.26 | 34.07 | 41.92 | 47.16 | **5.25** | 25.07 | 29.11 | 37.27 | 45.86 | 52.33 |
| **5.5** | 26.6 | 30.88 | 38.24 | 46.67 | 52.16 | **5.5** | 29.05 | 33.46 | 42.1 | 50.97 | 57.39 |
| **5.75** | 30.12 | 34.74 | 42.57 | 51.35 | 56.96 | **5.75** | 33.19 | 37.94 | 46.94 | 55.91 | 62.14 |
| **6** | 33.85 | 38.77 | 46.97 | 55.91 | 61.51 | **6** | 37.42 | 42.48 | 51.7 | 60.63 | 66.57 |
| **6.25** | 37.72 | 42.89 | 51.35 | 60.29 | 65.79 | **6.25** | 41.68 | 47.01 | 56.34 | 65.08 | 70.66 |
| **6.5** | 41.67 | 47.04 | 55.66 | 64.44 | 69.76 | **6.5** | 45.93 | 51.47 | 60.79 | 69.26 | 74.42 |
| **6.75** | 45.65 | 51.16 | 59.84 | 68.35 | 73.41 | **6.75** | 50.13 | 55.82 | 65.03 | 73.12 | 77.84 |
| **7** | 49.61 | 55.2 | 63.84 | 71.99 | 76.74 | **7** | 54.24 | 60.02 | 69.02 | 76.66 | 80.93 |
| **7.25** | 53.51 | 59.12 | 67.63 | 75.32 | 79.73 | **7.25** | 58.23 | 64.03 | 72.73 | 79.88 | 83.69 |
| **7.5** | 57.32 | 62.88 | 71.17 | 78.36 | 82.39 | **7.5** | 62.06 | 67.82 | 76.15 | 82.76 | 86.13 |
| **7.75** | 61 | 66.46 | 74.44 | 81.09 | 84.73 | **7.75** | 65.72 | 71.37 | 79.27 | 85.32 | 88.28 |
| **8** | 64.52 | 69.83 | 77.43 | 83.51 | 86.75 | **8** | 69.18 | 74.67 | 82.08 | 87.56 | 90.13 |
| **8.25** | 67.86 | 72.97 | 80.14 | 85.63 | 88.49 | **8.25** | 72.44 | 77.7 | 84.57 | 89.49 | 91.72 |
| **8.5** | 71.01 | 75.88 | 82.55 | 87.47 | 89.94 | **8.5** | 75.47 | 80.46 | 86.77 | 91.13 | 93.05 |
| **8.75** | 73.94 | 78.54 | 84.69 | 89.03 | 91.14 | **8.75** | 78.28 | 82.96 | 88.68 | 92.5 | 94.16 |
| **9** | 76.66 | 80.95 | 86.54 | 90.34 | 92.12 | **9** | 80.86 | 85.18 | 90.31 | 93.63 | 95.07 |
| **9.25** | 79.15 | 83.11 | 88.13 | 91.42 | 92.9 | **9.25** | 83.2 | 87.15 | 91.68 | 94.53 | 95.79 |
| **9.5** | 81.42 | 85.03 | 89.48 | 92.29 | 93.51 | **9.5** | 85.31 | 88.86 | 92.81 | 95.24 | 96.36 |
| **9.75** | 83.45 | 86.71 | 90.59 | 92.98 | 93.98 | **9.75** | 87.19 | 90.34 | 93.73 | 95.78 | 96.8 |
| **10** | 85.26 | 88.17 | 91.51 | 93.52 | 94.33 | **10** | 88.84 | 91.6 | 94.46 | 96.17 | 97.12 |
| **10.25** | 86.85 | 89.42 | 92.25 | 93.93 | 94.61 | **10.25** | 90.29 | 92.65 | 95.03 | 96.46 | 97.37 |
| **10.5** | 88.23 | 90.48 | 92.83 | 94.24 | 94.84 | **10.5** | 91.53 | 93.51 | 95.46 | 96.66 | 97.55 |
| **10.75** | 89.41 | 91.36 | 93.29 | 94.48 | 95.05 | **10.75** | 92.57 | 94.22 | 95.79 | 96.81 | 97.69 |
| **11** | 90.4 | 92.08 | 93.65 | 94.68 | 95.25 | **11** | 93.45 | 94.78 | 96.03 | 96.93 | 97.81 |
| **11.25** | 91.22 | 92.67 | 93.93 | 94.86 | 95.49 | **11.25** | 94.16 | 95.22 | 96.21 | 97.04 | 97.94 |
| **11.5** | 91.89 | 93.13 | 94.17 | 95.05 | 95.77 | **11.5** | 94.72 | 95.56 | 96.36 | 97.16 | 98.08 |
| **11.75** | 92.42 | 93.51 | 94.39 | 95.26 | 96.11 | **11.75** | 95.16 | 95.82 | 96.5 | 97.32 | 98.24 |
| **12** | 92.83 | 93.81 | 94.61 | 95.52 | 96.53 | **12** | 95.48 | 96.02 | 96.65 | 97.53 | 98.45 |
| **12.25** | 93.15 | 94.06 | 94.84 | 95.83 | 97.04 | **12.25** | 95.72 | 96.19 | 96.83 | 97.8 | 98.71 |
| **12.5** | 93.39 | 94.28 | 95.12 | 96.21 | 97.62 | **12.5** | 95.88 | 96.33 | 97.04 | 98.14 | 99.02 |
| **12.75** | 93.57 | 94.48 | 95.44 | 96.66 | 98.29 | **12.75** | 95.98 | 96.48 | 97.3 | 98.55 | 99.38 |
| **13** | 93.72 | 94.69 | 95.81 | 97.18 | 99.04 | **13** | 96.04 | 96.63 | 97.6 | 99.02 | 99.79 |
| **13.25** | 93.86 | 94.92 | 96.24 | 97.76 | 99.84 | **13.25** | 96.09 | 96.79 | 97.96 | 99.55 | 100 |
| **13.5** | 94.01 | 95.18 | 96.73 | 98.38 | 100 | **13.5** | 96.13 | 96.99 | 98.35 | 100 | 100 |
| **13.75** | 94.18 | 95.49 | 97.27 | 99.04 | 100 | **13.75** | 96.18 | 97.21 | 98.76 | 100 | 100 |
| **14** | 94.41 | 95.85 | 97.84 | 99.7 | 100 | **14** | 96.25 | 97.46 | 99.18 | 100 | 100 |
| **14.25** | 94.71 | 96.26 | 98.42 | 100 | 100 | **14.25** | 96.37 | 97.74 | 99.58 | 100 | 100 |
| **14.5** | 95.1 | 96.73 | 98.97 | 100 | 100 | **14.5** | 96.54 | 98.02 | 99.91 | 100 | 100 |
| **14.75** | 95.6 | 97.26 | 99.46 | 100 | 100 | **14.75** | 96.77 | 98.31 | 100 | 100 | 100 |
| **15** | 96.22 | 97.82 | 99.85 | 100 | 100 | **15** | 97.06 | 98.57 | 100 | 100 | 100 |
| **15.25** | 96.97 | 98.42 | 100 | 100 | 100 | **15.25** | 97.43 | 98.77 | 100 | 100 | 100 |
| **15.5** | 97.87 | 99.03 | 100 | 100 | 100 | **15.5** | 97.88 | 98.9 | 100 | 100 | 100 |
| **15.75** | 98.93 | 99.63 | 100 | 100 | 100 | **15.75** | 98.4 | 99.35 | 100 | 100 | 100 |
| **16** | 100 | 100 | 100 | 100 | 100 | **16** | 99 | 100 | 100 | 100 | 100 |
